# Genistein Reduces the Risk of Diabetes in Long-Term Hospitalized Schizophrenic Patients

**DOI:** 10.3390/bs16010021

**Published:** 2025-12-22

**Authors:** Yiying Sun, Bin Liu, Tingting Jiang, Yi Guo, Ying Xia, Zhicheng Cao, Haiping Fang, Yi Yang, Xirong Sun

**Affiliations:** Shanghai Pudong New Area Mental Health Center (Tongji University Affiliated Mental Health Center), Tongji University Clinical Research Center for Mental Disorders, Shanghai 200124, China; sunyy@shspdjw.com (Y.S.); liub@shspdjw.com (B.L.); jiangtt@shspdjw.com (T.J.); guoy@shspdjw.com (Y.G.); xiay@shspdjw.com (Y.X.); caozc@shspdjw.com (Z.C.); fanghp@shspdjw.com (H.F.)

**Keywords:** schizophrenia, diabetes mellitus, long-term hospitalization, SVM, MD

## Abstract

To identify clinical risk factors for diabetes mellitus (DM) in long-stay schizophrenia (LS-SCZ) patients, explore shared molecular mechanisms of schizophrenia and DM, and validate targeted interventions. Clinical data of LS-SCZ patients were analyzed via multiple logistic regression to identify DM risk factors. Differentially expressed genes (DEGs) from GSE53987/GSE161355 datasets were screened; overlapping DEGs were analyzed for co-expression and KEGG enrichment. Support vector machine (SVM) selected feature genes whose diagnostic efficacy was evaluated by ROC curves. Molecular docking verified Genistein’s binding with feature gene proteins. Risk factors for diabetes in LS-SCZ patients included age ≥50 years, hospitalization >20 years, BRI >5.306, family history of diabetes, and hypertension (all *p* < 0.05). Molecularly, 27 overlapping DEGs from two datasets were enriched in neuroactive ligand–receptor interaction, HIF-1, and MAPK pathways. SVM identified four feature genes (NPY, MKNK2, IFITM3, and S100A8) with good diagnostic efficacy. Genistein bound strongly to their proteins (binding energies: −8.98 to −6.026 kcal/mol). In conclusion, LS-SCZ patients have high DM risk. Targeting clinical risk factors and using Genistein for feature genes may reduce DM comorbidity.

## 1. Introduction

Schizophrenia is a chronic mental illness that is highly prevalent and difficult to treat. Its core characteristics are the interweaving of negative and positive symptoms, such as fragmented thinking, emotional indifference, hallucinations, and delusions. It not only seriously damages the patient’s social function and quality of life, but also poses a long-term threat to their physical health (Noort et al., 2024). Patients with schizophrenia (LS-SCZ) who are hospitalized for long-term treatment face more significant challenges in terms of their health management due to prolonged treatment cycles, limited space for activities, and a high dependence on medical care to regulate their daily rhythm. A fixed diet may result in insufficient dietary fiber intake and a high proportion of high-sugar and high-fat foods. The closed hospital environment significantly reduces daily exercise, and some antipsychotic drugs may cause metabolic disorders, which together increase the risk of comorbidities such as diabetes in this population (Mizuno et al., 2022; Rahme et al., 2023). Although schizophrenia and diabetes have been studied individually, few quantitative analyses have examined their interaction in comorbidity.

In Shanghai’s Pudong New Area, a region with concentrated medical resources and a diverse population structure, the scale and medical needs of long-term hospitalized patients with schizophrenia persist, yet their comorbidity with diabetes has not yet received sufficient attention. Existing studies have confirmed that the prevalence of diabetes in ordinary patients with schizophrenia is significantly higher than in the general population. Long-term hospitalized patients often find it difficult to detect abnormal blood sugar levels early due to their low level of disease awareness and limited self-health management capabilities. Once diagnosed with diabetes, they are more likely to develop serious complications such as diabetic nephropathy and neuropathy, which not only exacerbate the patient’s physical and mental suffering, but also prolong hospitalization time, increase the medical burden, and even lead directly to increased all-cause mortality and shortened life expectancy (Chan et al., 2021; Huo et al., 2021). Although clinical observation has confirmed that the risk of comorbidity of diabetes (DM) in LS-SCZ is significantly increased, and some clinical risk factors (such as age and body index abnormalities) have been preliminarily identified, the molecular mechanism of the comorbidity of the two is still unclear, which limits the development of precise intervention strategies. Current research focuses on the gene regulation or pathway abnormalities of a single disease, lacking systematic exploration of shared molecular characteristics of schizophrenia and diabetes. Meanwhile, the potential intervention methods based on molecular targets, such as low-toxicity natural compounds, urgently need to be experimentally validated for their application value in this comorbidity scenario.

In view of this, this study takes LS-SCZ patients who have been hospitalized for more than one year in the Pudong New Area of Shanghai as the subjects; adopts a retrospective cohort design; collects their demographic, hospitalization, and laboratory data; and focuses on the analysis of the relationships between age, length of hospitalization (especially >20 years), combined hypertension, BMI, and antipsychotic drug use and diabetes, while paying attention to the metabolic risk impact of BRI > 5.306. Furthermore, by combining gene chip data to screen for overlapping differentially expressed genes in two types of diseases, core feature genes were identified through pathway enrichment and machine learning, and potential intervention compounds were validated through molecular docking. The purpose of this study is to determine the prevalence of comorbidity and key influencing factors of diabetes in this population, and to provide a basis for developing personalized prevention strategies and improving patient prognosis, as well as a reference for research in other regions.

## 2. Subjects and Methods

### 2.1. Study Subjects

A total of 489 patients with long-term schizophrenia (LS-SCZ) hospitalized at the Pudong New Area Mental Health Center (hospitalized for more than one year) who responded to a questionnaire survey were enrolled as study subjects. Ethical Considerations: This study was approved by the Ethics Committee of the Shanghai Pudong New Area Mental Health Center (PDJWLL2023024).

Inclusion Criteria: ① Patients who had been hospitalized at the Shanghai Pudong New Area Mental Health Center for more than one year as of 31 December 2024; ② patients who met the diagnostic criteria for schizophrenia according to the International Statistical Classification of Diseases and Related Health Problems (10th Revision) (ICD-10); and ③ patients with schizophrenia were in a stable phase, had self-awareness and behavioral competence, and had received informed consent from the patient and guardian and signed a consent form.

Exclusion criteria: ① Missing essential research information, such as no blood sugar data or an inability to provide accurate height and weight information due to prolonged bed rest; ② under 18 years of age; ③ unable to cooperate with study follow-up; and ④ subjects who should be excluded after discussion by the research team, such as those with abnormal data that could not be traced and corrected.

### 2.2. Research Methods

A standardized epidemiological questionnaire was administered by trained interviewers. The survey collected general demographic information, histories of mental illness and medication use, previous and family history of diabetes, and major diabetes risk factors. Additionally, trained interviewers used standardized, calibrated instruments to measure the height, weight, waist circumference, hip circumference, and blood sugar levels of participants. The Chinese Adult Body Shape Index (ABSI) (higher values generally indicate higher health risks associated with central obesity) and Body Roundness Index (BRI) were calculated.

### 2.3. Definition of Indicators

Schizophrenia diagnosis was based on a confirmed diagnosis verified in the HIS system. Diabetes diagnosis was established by a township (district) or higher-level hospital. Whether the patient developed diabetes during hospitalization is determined based on the time of admission and the time of diabetes diagnosis. The diagnosis of diabetes is based on the guidelines of the American Diabetes Association. The ABSI uses the modified Chinese Adult Body Shape Index. The BRI is a new body index proposed by Thomas et al. in 2013 to determine abdominal obesity based on body weight and height (Thomas et al., 2013).

### 2.4. Quality Control

Full-process quality control was implemented. The survey was completed by uniformly trained medical professionals. Calibrated instruments were used for relevant measurements. A dedicated person reviews and checks 20% of the questionnaires daily, and the questionnaire compliance rate reaches 95%. The data are then double-entered and proofread.

### 2.5. Dataset Screening and Pathway Enrichment Analysis

Based on the GEO database (https://www.ncbi.nlm.nih.gov/geo/ (accessed on 7 September 2025)), obtain datasets GSE53987 and GSE161355. Dataset GSE53987 includes 48 patients with schizophrenia and 55 healthy controls. Dataset GSE161355 includes 18 diabetes patients and 15 controls. The “limma” R software package v.3.66.0 was used to screen for differentially expressed genes. The “clusterProfiler” R package v.3.10.1 was used for GO and KEGG analyses.

### 2.6. Machine Learning SVM Screening of Feature Genes, Construction of ROC Curve, and Molecular Docking (MD)

Construct a linear kernel SVM model using “caret and kernlab” R packages, recursively sort and remove the least important genes based on feature weights, and combine cross-validation to determine the optimal set of base factors while maintaining the highest discriminative performance. Subsequently, to quantitatively evaluate the diagnostic efficacy of the feature gene set, based on its prediction results on the test set, the “pROC” package was used to draw the working characteristic curve of the subjects and calculate the area under the curve. Obtain the three-dimensional structure of the protein from the PDB database and perform preprocessing such as hydrogenation and charge addition. Optimize the potential active small-molecule ligands for energy, and then use the “AutoDock Vina” program v1.2.x to perform docking simulations within the defined active pocket. Finally, select the optimal binding conformation based on the binding free energy, and analyze key interaction forces such as hydrogen bonding and hydrophobic interactions to elucidate possible binding modes.

### 2.7. Statistical Analysis

EpiData 3.1 software was used for the double entry of data, and SPSS 20.0 software was used for statistical analysis. The data were described using frequency (constituent ratio, %), and the χ^2^ test was used for statistical testing. The prevalence rate was standardized based on the population composition of the Seventh National Population Census. Multivariate logistic regression was used for multivariate analysis.

## 3. Results

### 3.1. Diabetes Prevalence

A total of 489 patients with LS-SCZ were surveyed, and 489 valid questionnaires were collected. Among them, 121 patients were diagnosed with type 2 diabetes (T2DM), while 368 were undiagnosed. Ninety LS-SCZ patients experienced a gradual increase in blood glucose levels starting 3–4 years after admission and were subsequently diagnosed with diabetes. The average age of diagnosis was 6.30 ± 3.56 years after admission. There were no statistically significant differences in gender or age compared with patients diagnosed with diabetes before admission (*p* > 0.05), but there was a statistically significant difference in the age of diabetes onset (χ^2^ = 41.835, *p* < 0.05) (Table 1 and Table 2).

### 3.2. Major Risk Factors for Diabetes

A comparative analysis demonstrated that LS-SCZ + T2DM patients differed significantly from their non-diabetic LS-SCZ counterparts across six clinical variables, namely age, hospitalization duration, BRI, diabetes family history, presence of hypertension, and daily sedentary duration (all *p* < 0.05). Notably, no significant inter-group variation was detected in the ABSI, suggesting this index may not be associated with T2DM comorbidity in the LS-SCZ population (*p* > 0.05) (Table 3).

### 3.3. Multivariate Analysis

Taking diabetes status as the dependent variable, we constructed a regression model incorporating age group, hospitalization duration, BRI, family history of diabetes, and daily sedentary time as predictor variables. The results revealed that age ranging from 50 to 70 years, hospitalization duration exceeding 20 years, BRI value greater than 5.306, positive family history of diabetes, and comorbid hypertension were identified as independent risk factors for diabetes mellitus in LS-SCZ patients (all *p* < 0.05) (Table 4).

### 3.4. Screening of Schizophrenia- and Diabetes-Related Genes and Enrichment Analysis of Their Pathways

**Figure 1:**
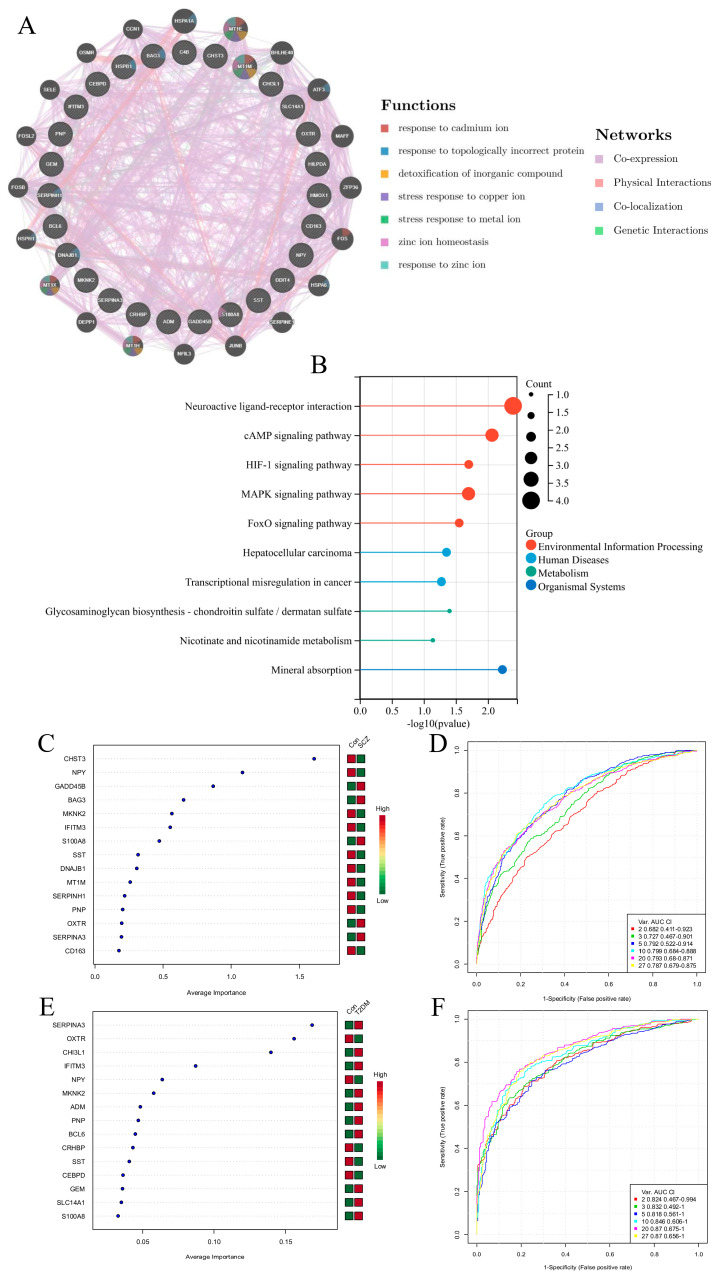
Screening of schizophrenia- and diabetes-related genes and enrichment analysis of their pathways. (**A**) Gene co expression analysis. (**B**) KEGG pathway enrichment analysis of overlapping genes. (**C**,**D**) SVM-based screening of feature genes for GSE53987. (**E**,**F**) SVM-based screening of feature genes for GSE161355.

As shown in Figure S1, a total of 130 differentially expressed genes (107 upregulated and 23 downregulated) were screened based on the GSE53987 dataset. The enrichment analysis of upregulated gene pathways mainly focused on the HIF-1 signaling pathway, the TNF signaling pathway, the Ferroptosis and NF-kappa B signaling pathway, etc. As shown in Figure S2, based on the GSE161355 dataset, a total of 821 differentially expressed genes were screened (661 upregulated and 160 downregulated). The enrichment analysis of upregulated gene pathways mainly focuses on the AGE-RAGE signaling pathway in diabetes and apoptosis, and the VEGF signaling pathway. In order to explore the comorbidity characteristics of schizophrenia and diabetes, we crossed their different genes and obtained 27 overlapping genes. In total, 20 co-expressed genes were obtained through gene co-expression analysis of these overlapping genes (Figure 1A). KEGG pathway enrichment analysis showed that overlapping genes are mainly enriched in neuroactive ligand–receptor interaction, the cAMP signaling pathway, the HIF-1 signaling pathway, the MAPK signaling pathway, the FoxO signaling pathway, etc. (Figure 1B). As shown in Figure 1C–F, the overlapping genes were further screened using SVM and sorted by importance. The top 10 genes in the two datasets were intersected to obtain four feature genes (NPY, MKNK2, IFITM3, and S100A8).

### 3.5. Diagnostic Evaluation and Molecular Docking of Characteristic Genes

**Figure 2:**
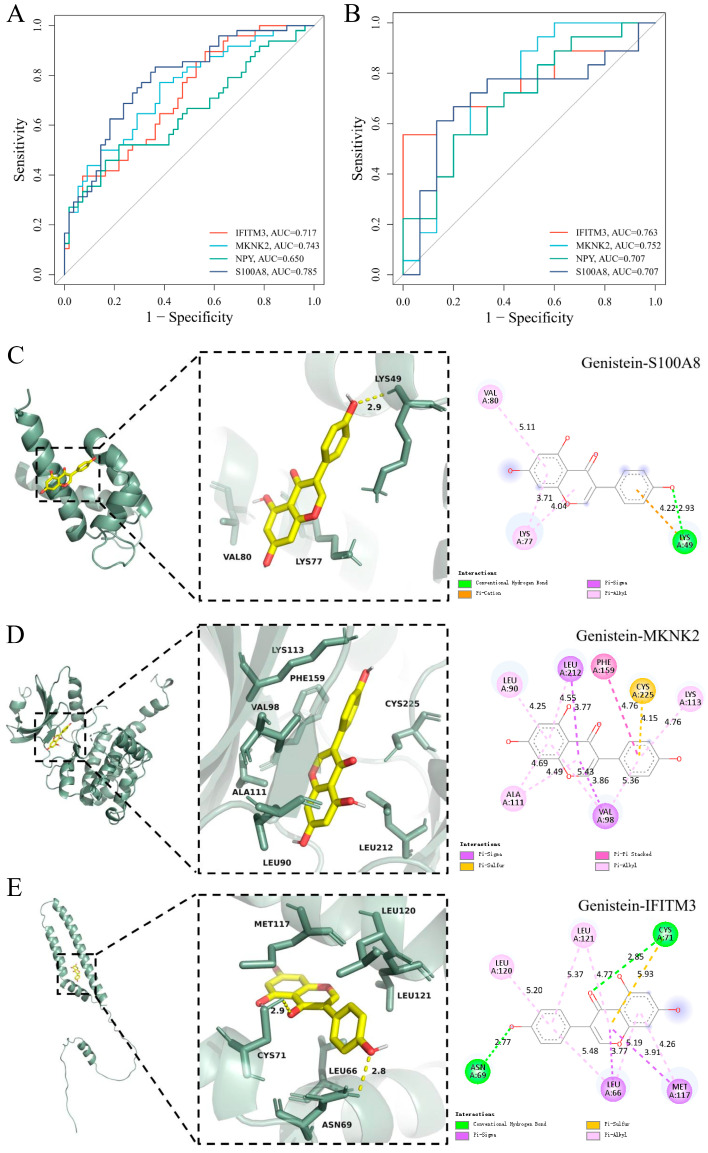
Diagnostic evaluation and molecular docking of characteristic genes. (**A**) ROC curve of characteristic genes based on GSE53987. (**B**) ROC curve of characteristic genes based on GSE161355. (**C**) Genistein–MKNK2. (**D**) Genistein–IFITM3. (**E**) Genistein–S100A8.

The diagnostic efficacy of feature genes in the two diseases was evaluated based on ROC curves, and the results are shown in Figure 2A,B. All four feature genes have good diagnostic efficacy in both diseases. In order to screen targeted interventions based on feature genes, we identified the top 10 interventions (Table S1), among which Genistein is a natural compound with low toxicity and low side effects. As shown in Figure 2C–E, the binding energies of Genistein with MKNK2 (8XFM), IFITM3 (AF-Q01628), and S100A8 (4GGF) were −8.98 kcal/mol, −6.852 kcal/mol, and −6.026 kcal/mol, respectively, all less than −5 kcal/mol, indicating the strong binding ability of Genistein with characteristic genes.

## 4. Discussion

### 4.1. Schizophrenia: Core Clinical Manifestations, Multidimensional Impairments, and Impact on Quality of Life

Schizophrenia is a severe chronic mental disorder with a high incidence worldwide (*The Bearing of the Joint Family Unit on the Diagnosis and Delay of Schizophrenia Treatment*, 2023). Its core clinical features include not only positive symptoms such as hallucinations and delusions, negative symptoms such as apathy and social withdrawal, but also significant cognitive dysfunction, such as distractibility, impaired executive function, and slowed information processing speed (Kwon et al., 2026; Lim et al., 2026). These multidimensional pathological manifestations collectively lead to the severe impairment of patients’ social functioning. Patients not only struggle to engage in normal work, study, and interpersonal communication, but also experience a diminished capacity to manage their own health. Consequently, they are at a disadvantage in terms of the prevention, identification, and management of physical illnesses, particularly metabolic diseases. Ultimately, this creates a vicious cycle of “mental symptoms–social function loss–physical health deterioration”, significantly reducing patients’ quality of life (Helaly & Ghorab, 2023; Mitchell et al., 2013).

### 4.2. Diabetes Mellitus: Core Pathophysiology, Clinical Manifestations, Multi-System Complications, and Health Risks

Diabetes mellitus is one of the most burdensome chronic metabolic diseases worldwide. Its core pathological mechanism involves the disruption of glucose and lipid metabolism caused by insufficient insulin secretion or decreased insulin sensitivity of target cells (Agarwal et al., 2017; Dai et al., 2026). Although the “three mores and one less” (excessive drinking, excessive eating, excessive urination, and weight loss) are its typical clinical manifestations, approximately 30% to 40% of patients with type 2 diabetes mellitus (T2DM) exhibit no obvious symptoms in the early stages and are only incidentally diagnosed with abnormal glycemic levels during physical examinations or complication screening (Anderson & Leahy, 2025; Brewster et al., 2020; Hsieh & Hsieh, 2021). Furthermore, long-term poor blood sugar control may cause multi-system complications: retinopathy can lead to progressive vision loss or even blindness (Gonzalez et al., 2021; Schreur et al., 2021), diabetic nephropathy is the leading cause of end-stage renal disease (Miraghajani et al., 2019; Punaro et al., 2019), and peripheral neuropathy can result in limb numbness, pain, and even ulceration and necrosis (Lai et al., 2020; Malakoti et al., 2024; Raafat et al., 2021). These complications not only significantly increase the medical burden on patients but are also directly associated with an increased risk of malignant tumors such as cardiovascular disease, pancreatic cancer, and colorectal cancer, posing a serious threat to patient life expectancy (Alam et al., 2018).

### 4.3. Prevalence, Underlying Mechanisms, Risk Factors, and Clinical Monitoring Gaps of T2DM in Patients with Severe Mental Disorders

The risk of developing T2DM is significantly different in patients with psychiatric disorders. Our research is consistent with many previous studies, which reported that the prevalence of type 2 diabetes in patients with severe mental disorders (especially schizophrenia) is 2–3 times higher than that in the general population (Cannon et al., 2024; Chen et al., 2014; John et al., 2024; Schoepf et al., 2014). This difference is not only related to lifestyle changes caused by the disease itself (such as irregular diet and reduced activity), but also the metabolic effects of antipsychotic drugs, which are a key driving factor. Among these, second-generation antipsychotic drugs (such as olanzapine, risperidone, and quetiapine) were effective in terms of improving mental symptoms (X. Chang et al., 2023; Fauska et al., 2024). Although second-generation antipsychotics (such as olanzapine and risperidone) are widely used for symptom control in long-term hospitalized patients with schizophrenia, their metabolic risks can vary depending on dosage, duration of treatment, and drug-specific characteristics. For example, compared to aripiprazole, high-dose or long-term use of olanzapine is associated with more significant weight gain and insulin resistance (Burschinski et al., 2023; X. Liu et al., 2017). Unfortunately, due to the retrospective design of this study, the existing dataset lacks detailed records of individual patients’ antipsychotic drug dosage, treatment duration, and history of medication administration, making it impossible to distinguish drug-specific metabolic risks as an independent confounding factor in the analysis. In addition, some drugs can activate the hypothalamic appetite regulation pathway and inhibit lipase activity, leading to hyperphagia and abdominal obesity in patients. The inflammatory response of adipocytes caused by abdominal obesity (such as increased secretion of TNF-α and IL-6) (Mani & Alshammeri, 2023; Shao et al., 2015; Turra et al., 2020) further disrupts the insulin signaling pathway, reducing the sensitivity of target organs, including skeletal muscle and adipose tissue to insulin, and ultimately inducing or exacerbating insulin resistance (Scheuer et al., 2022). Notably, metabolic monitoring of patients with schizophrenia in clinical practice often prioritizes psychiatric symptoms over physical health. Most medical institutions perform blood glucose testing only when patients exhibit symptoms such as excessive thirst or sudden weight gain. Regular glucose tolerance testing (OGTT) and glycated hemoglobin (HbA1c) screening coverage for asymptomatic patients is less than 50%, resulting in many prediabetic patients missing opportunities for early intervention and eventually developing diagnosed diabetes. This study revealed a crude diabetes prevalence of 24.74% (15.65% after standardization) in LS-SCZ patients, significantly higher than the prevalence of diabetes in the general Chinese population reported by the International Diabetes Federation (IDF) (10.6%) and the Chinese Medical Association’s Endocrinology Section (10.6%). Blood glucose monitoring revealed that, beginning in the fourth year, blood glucose levels gradually increased, reaching levels comparable to those in the pre-hospitalized diabetes group, despite the absence of HbA1c and glucose tolerance testing. These findings suggest that intervention against and monitoring of prediabetes should be intensified for patients with occasional abnormal blood glucose levels to ensure early detection and treatment, thereby reducing or delaying the onset of diabetic complications. Retrospective study and multivariate logistic regression analysis identified age 50 to 70 years, hospitalization for more than 20 years, BRI greater than 5.306, family history, and comorbid hypertension were risk factors for diabetes in LS-SCZ patients. This study found that the prevalence of T2DM was significantly higher in long-term hospitalized patients with schizophrenia than in the general population, and the lack of standardized metabolic monitoring was a significant contributing factor. Neglect of patients’ physical health in clinical practice, coupled with patients’ weak self-management abilities, exacerbated the risk of metabolic diseases. From the perspective of self-determination theory, patients’ needs for autonomy, competence, and social connection were not fully met due to the limitations imposed by their psychiatric symptoms and hospital environment. This further weakened their intrinsic motivation to participate in health management, creating a cycle of ‘lack of motivation–negative behavior–deteriorating health’. This suggests that clinical practice needs to strengthen metabolic health interventions for this group. Based on standardized monitoring, empowering guidance should be provided to meet patients’ core psychological needs, thereby improving their self-management skills and forming a closed-loop intervention from both theoretical and practical perspectives.

### 4.4. Molecular Mechanisms and Targeted Intervention Potential of Comorbid Schizophrenia and Diabetes Mellitus During Long-Term Hospitalization

This study expands the understanding of the comorbidity mechanism of LS-SCZ and T2DM at the molecular level. Its core findings are highly consistent with existing research results, and at the same time provide a new theoretical anchor for the precise diagnosis and targeted intervention of this comorbidity. Gene expression analysis revealed that differentially expressed genes (DEGs) screened from the GSE53987 dataset were significantly enriched in the HIF-1 and TNF signaling pathways. This is consistent with previous findings—abnormal activation of the HIF-1 pathway can exacerbate insulin resistance (IR) by regulating key glycolytic enzymes (such as HK2 and PFK1), while TNF-α-mediated chronic low-grade inflammation is a core bridge connecting neuroinflammation in schizophrenia and metabolic disorders like type 2 diabetes mellitus (T2DM) (Baryła et al., 2022; Y. Liu et al., 2013). The AGE-RAGE signaling pathway enriched in DEGs from the GSE161355 dataset is a classic pathway in the pathological process of T2DM. Its overactivation can exacerbate vascular damage and neuronal apoptosis by inducing oxidative stress and activating NF-κB. This also explains why LS-SCZ patients often have more severe glucose metabolism abnormalities and neurological function decline.

The 27 overlapping DEGs were further enriched in neuroactive ligand–receptor interaction, MAPK, and FoxO signaling pathways. This result reveals the molecular coupling mechanism of the “psycho-metabolic comorbidity”: abnormalities in the neuroactive ligand–receptor interaction pathway (such as the imbalance of dopamine and serotonin receptor expression) are not only a core pathological feature of schizophrenia, but can also affect insulin sensitivity by regulating the hypothalamic–pituitary–adrenal axis (Y. Liu et al., 2013); meanwhile, persistent activation of the MAPK pathway can simultaneously mediate neurocytotoxicity and pancreatic β-cell dysfunction, and inactivation of the FoxO pathway weakens its regulatory role in glucose homeostasis and neuroprotective function (Cho, 2024). These three factors together constitute the molecular network basis for the comorbidity of the two diseases.

Four characteristic genes (NPY, MKNK2, IFITM3, and S100A8) selected by machine learning all demonstrated excellent diagnostic efficacy for dual pathologic effects. Their pleiotropic biological functions provide a key explanation for the comorbidity mechanism: NPY, as a neuropeptide hormone, not only participates in the pathogenesis of T2DM by regulating appetite and energy metabolism (its downregulation can lead to abnormal satiety and increased risk of obesity), but also exacerbates cognitive impairment in schizophrenia by affecting the activity of prefrontal cortex neurons (Gragnoli et al., 2016); MKNK2, as a downstream effector molecule of the MAPK pathway, can promote the transcription of pro-inflammatory factors (such as IL-6 and IL-1β) through phosphorylation of eIF4E, while inhibiting the activation of IRS-1 in the insulin signaling pathway, forming a vicious cycle of “inflammation–IR” (Wang et al., 2013); and IFITM3 and S100A8 are core markers of innate immunity and inflammatory responses, with the former affecting pancreatic β-cells by regulating autophagy. Cell survival is crucial, as the latter, as a damage-associated molecular pattern (DAMP) molecule, can activate the TLR4/NF-κB pathway, exacerbating neuroinflammation in schizophrenia and accelerating pancreatic islet function decline in type 2 diabetes mellitus (Sanders et al., 2017; Shamir et al., 2023).

The key value of molecular docking experiments lies in the fact that the natural isoflavone compound Genistein possesses highly efficient targeted binding capabilities for each of the target proteins. Previous studies have confirmed that Genistein can downregulate inflammatory pathways by inhibiting tyrosine kinase activity and improve insulin sensitivity by activating the AMPK pathway (Guevara-Cruz et al., 2020). This study is the first to associate its target with the core genes of LS-SCZ-T2DM comorbidity, providing a molecular basis for “multi-target synergistic intervention”—its binding to MKNK2 may block the MAPK-eIF4E inflammatory axis, its binding to IFITM3 can regulate autophagy homeostasis, and its binding to S100A8 can inhibit TLR4-mediated neuro-metabolic inflammation. These three factors together constitute the potential mechanism of Genistein for comorbidity intervention.

### 4.5. Limitations of the Study on T2DM Comorbidity in Long-Stay Patients with Schizophrenia and Future Research Prospects

The limitations of this study were as follows: (1) LS-SCZ patients received multiple antipsychotics over extended periods during hospitalization, and the study failed to analyze the metabolic effects of different antipsychotics on patients. (2) The patients’ conditions prior to the onset of diabetes were not included in the analysis. The patients’ medication history after admission could be recorded to explore the effects of antipsychotics on the onset of T2DM. (3) The study results were consistent with previous studies (Y. Chang et al., 2015), and no correlation was confirmed between the ABSI and diabetes in LS-SCZ patients. (4) Although this study identified the key risk factors for diabetes in LS-SCZ patients, the control design has limitations—it primarily used internal patient controls and lacked large-scale on-site controls of the general population in Shanghai. In the future, multicenter matched controls can be used to include multi-dimensional controls such as a normal population and non-hospitalized patients with schizophrenia; molecular biological indicators can be combined to deepen the mechanism analysis and enhance the research value. (5) The heterogeneity of samples in public datasets may affect the stability of DEG screening, and in vitro molecular docking does not take into account the effects of in vivo pharmacokinetics and post-translational protein modifications. Future research needs to validate the expression differences in characteristic genes using clinical samples and evaluate the intervention effect of Genistein in an animal model of LS-SCZ combined with T2DM. Furthermore, combined with transcriptome sequencing and metabolomics analysis, its mechanism of action in regulating comorbidity should be further elucidated. In addition, based on the above research, we have the following three recommendations. First, for modifiable lifestyle and metabolic risk factors, implement structured physical activity programs tailored to LS-SCZ patients’ cognitive and functional status—e.g., 30 min of daily supervised group activities (brisk walking, chair yoga, or low-intensity resistance training)—with adherence enhanced via pedometer/activity log tracking. Second, address metabolic screening gaps with a stratified monitoring protocol: high-risk patients (age ≥ 50 years, BRI > 5.306, comorbid hypertension) undergo quarterly fasting blood glucose/HbA1c tests and annual OGTT (even asymptomatic); those with a diabetes family history receive additional insulin resistance screening for early prediabetes detection. Embed these protocols into routine psychiatric care, with designated staff tracking abnormal results for timely endocrinology referral. Third, mitigate long-term hospitalization-related metabolic risks by establishing a multidisciplinary care team. The team collaboratively adjusts antipsychotic regimens (prioritizing metabolically neutral agents like aripiprazole for high-risk patients when appropriate), develops personalized diets, and delivers simplified health education (diabetes prevention lectures and visual blood glucose self-monitoring guidance) to improve health literacy—critical for patients with limited access to community health resources.

In summary, this study makes explicit contributions to both theoretical and practical domains regarding type 2 diabetes mellitus (T2DM) comorbidity in long-stay schizophrenia (LS-SCZ) patients. Theoretically, it confirms that the unique long-term hospitalization lifestyle of LS-SCZ patients—coupled with antipsychotic-induced metabolic effects—alters the natural progression of T2DM, leading to a significantly higher prevalence compared to the general population. This finding enriches the existing understanding of bidirectional interactions between severe mental disorders and metabolic diseases, providing empirical evidence for the “mental–physical comorbidity” theoretical framework. Practically, the study identifies key gaps in current clinical practice and offers actionable insights for optimizing T2DM prevention and management in this high-risk group.

## Figures and Tables

**Table 1:** Incidence of diabetes in LS-SCZ + T2DM patients (n = 121).

Basic Information		T2DM-B * (n = 31)	T2DM-A ^#^ (n = 90)	χ^2^	*p*
Gender	Male	17 (54.84)	54 (60.00)	0.253	0.615
	Woman	14 (45.16)	36 (40.00)		
Age	30~	0 (0.00)	2 (2.22)	1.541	0.819
	40~	1 (3.03)	4 (4.44)		
	50~	7 (22.58)	30 (33.33)		
	60~	12 (38.71)	38 (42.22)		
	≥70	11 (35.48)	16 (17.78)		
Age of onset of diabetes (years)	30~	10 (32.26)	3 (3.33)	41.835	<0.001
	40~	11 (35.48)	7 (7.78)		
	50~	7 (22.58)	32 (35.56)		
	60~	3 (9.68)	34 (37.78)		
	≥70	0 (0)	14 (15.56)		

Note: * T2DM-B: T2DM diagnosed before hospitalization. ^#^ T2DM-A: T2DM diagnosed after hospitalization.

**Table 2:** Blood Glucose Testing After Admission in Patients with LS-SCZ + T2DM and LS-SCZ (n = 489) (mmol/L).

Years of Hospitalization	T2DM-B * (n = 31)	T2DM-A ^#^ (n = 90)	LS-SCZ (n = 368)
0~1	7.06 ± 1.85	5.83 ± 1.02	5.01 ± 0.62
1~	6.96 ± 1.46	5.96 ± 1.09	4.95 ± 0.55
2~	7.13 ± 1.48	5.95 ± 1.02	4.96 ± 0.55
3~	6.98 ± 1.86	6.27 ± 1.39	4.94 ± 0.62
4~	6.79 ± 1.55	6.52 ± 1.6	4.98 ± 0.61
5~	6.60 ± 1.24	6.66 ± 1.83	5.01 ± 0.59
6~	6.96 ± 1.88	6.46 ± 1.47	5.00 ± 0.53
11~	6.91 ± 1.71	6.58 ± 1.44	5.09 ± 0.55
16~	6.99 ± 1.63	6.53 ± 1.65	5.23 ± 0.64
21~	6.56 ± 1.41	6.97 ± 1.65	5.10 ± 0.58

Note: * T2DM-B: T2DM diagnosed before hospitalization. ^#^ T2DM-A: T2DM diagnosed after hospitalization.

**Table 3:** Comparison of Risk Factors Between LS-SCZ + T2DM and LS-SCZ Patients (n = 489).

Basic Information		LS-SCZ + T2DM (n = 121)	LS-SCZ (n = 368)	χ^2^	*p*
Age	≤39	2 (1.65)	41 (11.14)	22.244	<0.001
	40~	8 (6.61)	66 (17.93)		
	50~	42 (34.71)	98 (26.63)		
	60~	48 (39.67)	112 (30.43)		
	≥70	21 (17.36)	51 (13.86)		
Duration of hospitalization	1~	5 (4.13)	26 (7.07)	46.258	<0.001
	5~	28 (23.14)	103 (27.99)		
	10~	31 (25.62)	171 (46.47)		
	15~	34 (28.10)	52 (14.13)		
	≥20	23 (19.01)	16 (4.35)		
ABSI	Q1 (<0.072)	25 (20.66)	98 (26.63)	2.868	0.412
	Q2 (0.072~0.076)	28 (23.14)	94 (25.54)		
	Q3 (0.077~0.081)	33 (27.27)	89 (24.18)		
	Q4 (>0.081)	35 (28.93)	87 (23.64)		
BRI	Q1 (<3.475)	20 (16.53)	103 (27.99)	13.601	0.004
	Q2 (3.475~4.324)	24 (19.83)	98 (26.63)		
	Q3 (4.325~5.306)	35 (28.93)	87 (23.64)		
	Q4 (>5.306)	42 (34.71)	80 (21.74)		
Family history of diabetes	No	112 (92.56)	363 (98.64)	12.101	0.001
	Yes	9 (7.44)	5 (1.36)		
Comorbidities of hypertension	No	67 (55.37)	302 (82.07)	35.038	<0.001
	Yes	54 (44.63)	66 (17.93)		
Daily meditation time	<8	86 (71.07)	298 (80.98)	5.297	0.021
	≥8	35 (28.93)	70 (19.02)		

**Table 4:** Multivariate logistic regression analysis of risk factors in LS-SCZ + T2DM and LS-SCZ patients (n = 489).

Factors		Wald	OR (95%CI)	*p*
Age	≤39	13.058	1.000	
	40~	0.521	1.86 (0.345~10.676)	0.470
	50~	6.341	7.056 (1.542~32.29)	0.012
	60~	4.947	5.638 (1.228~25.882)	0.026
	≥70	3.261	4.339 (0.882~21.343)	0.071
Duration of hospitalization	1~	34.289	1.000	
	5~	0.005	0.959 (0.313~2.943)	0.942
	10~	0.562	0.656 (0.218~1.974)	0.453
	15~	1.832	2.183 (0.705~6.759)	0.176
	≥20	7.984	5.978 (1.729~20.665)	0.005
BRI	Q1 (<3.475)	7.915		
	Q2 (3.475~4.324)	0.001	1.013 (0.487~2.108)	0.973
	Q3 (4.325~5.306)	3.116	1.862 (0.934~3.712)	0.078
	Q4 (>5.306)	4.631	2.129 (1.07~4.238)	0.031
Family history of diabetes	No		1.000	
	Yes	10.171	9.314 (2.363~36.704)	0.001
Comorbidities of hypertension	No		1.000	
	Yes	15.188	2.798 (1.668~4.693)	<0.001
Daily meditation time	<8		1.000	
	≥8	3.442	1.673 (0.971~2.883)	0.064

## Data Availability

The raw data supporting the conclusions of this article will be made available by the authors on request.

## Supplementary Materials

The following supporting information can be downloaded at: https://www.mdpi.com/article/10.3390/bs16010021/s1, Table S1. Top 10 targeted intervention agents. Figure S1. Screening and pathway enrichment analysis of differentially expressed genes related to schizophrenia. Figure S2. Screening and pathway enrichment analysis of diabetes related differentially expressed genes.
