# Supplementary material for: Genistein Reduces the Risk of Diabetes in Long-Term Hospitalized Schizophrenic Patients
Source: Behav Sci (Basel). 2025 Dec 22;16(1):21. doi: 10.3390/bs16010021 (PMC12837747; doi:10.3390/bs16010021)
Supplement: Supplementary file 1 [file behavsci-16-00021-s001.zip › behavsci-3955775-supplementary.pdf]

Table S1 Top 10 targeted intervention agents

Term	P-value	Adjusted P-value	Odds Ratio	Combined Score
norepinephrine	3.96E-5	0.008	398.92	4043.246
genistein	8.87E-4	0.053	45.85	322.184
Decitabine	0.003	0.053	30.382	179.525
scopolamine	0.003	0.053	43.239	251.284
pregnenolone	0.003	0.053	43.044	249.779
celecoxib	0.005	0.053	289.464	1545.991
Enkephalin	0.005	0.053	277.389	1470.198
XMD-12	0.005	0.053	266.28	1400.895
GSK650394A	0.006	0.053	246.531	1278.762
Dynorphin A porcine	0.006	0.053	246.531	1278.762

Screening and pathway enrichment analysis of genes related to schizophrenia

As shown in Figure S1, a total of 130 differentially expressed genes (107 upregulated and 23 downregulated) were screened based on the GSE53987 dataset. The enrichment analysis of upregulated gene pathways mainly focused on HIF-1 signaling pathway、TNF signaling pathway、PPAR signaling pathway、Ferroptosis、NF-kappa B signaling pathway And apoptosis, etc.

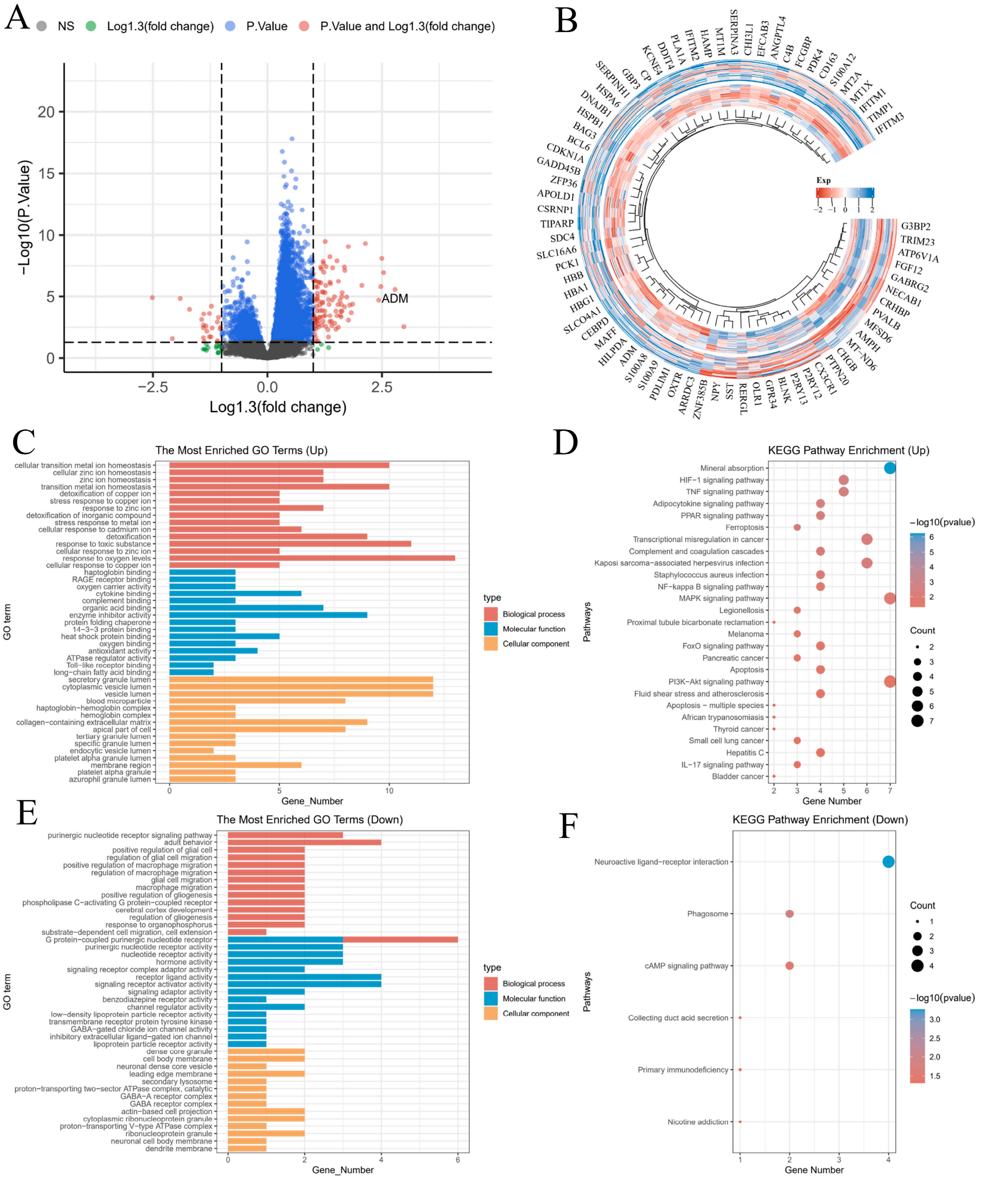

Figure S1 Screening and pathway enrichment analysis of differentially expressed genes related to schizophrenia

A, B: Differential genes in the GSE53987 dataset. C-F: GO and KEGG analysis of differentially expressed genes.

Screening of diabetes related genes and enrichment analysis of their pathways

As shown in Figure S2, based on the GSE161355 dataset, a total of 821 differentially expressed genes

were screened (661 up-regulated and 160 down regulated). The enrichment analysis of upregulated gene pathways mainly focuses on AGE-RAGE signaling pathway in diabetes, apoptosis, and VEGF signaling pathway.

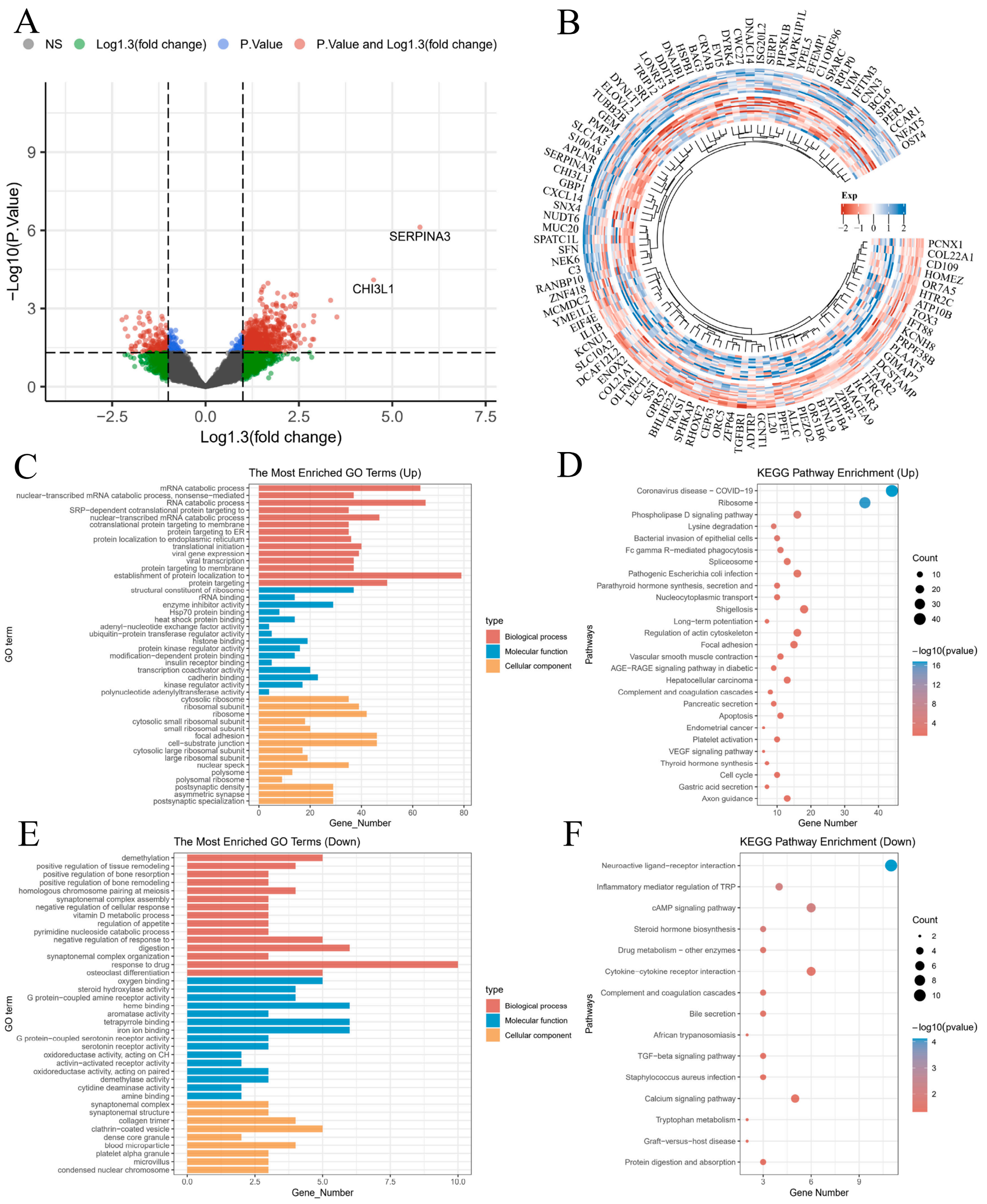

Figure S2 Screening and pathway enrichment analysis of diabetes related differentially expressed genes A. B: Differential genes in the GSE161355 dataset. C-F: GO and KEGG analysis of differentially expressed genes.
